# Management of acute sickle cell priapism in an African (Togo) pediatric department includes conservative measures and intracavernous epinephrine which is safe and efficacious

**DOI:** 10.1002/jha2.440

**Published:** 2022-04-26

**Authors:** Koffi Mawuse Guedenon, Mawouto Fiawoo, Djatougbe Ayaovi Elie Akolly, Etse Akpako, Balakibawi Esso, Fidèle Comlan Dossou, Adama Dodji Gbadoe

**Affiliations:** ^1^ Department of Pediatrics CHU Sylvanus Olympio, University of Lomé Togo; ^2^ University of Maryland Medical Systems/Midtown Campus Baltimore Maryland USA

**Keywords:** epinephrine, priapism, sickle cell (patient)

## Abstract

Priapism is a well‐known urologic complication of sickle cell anemia. This study describes the results of a protocol for the treatment of acute priapism by intracavernous injection of epinephrine due to unavailability of etilefrine. A descriptive cross‐sectional study of 18 cases of acute priapism in sickle cell patients treated in the pediatric department of the Sylvanus Olympio CHU from January 1 to December 31, 2020. The average age was 21.7 ± 7.7 years, the youngest patient was 8 and the oldest was 32 years old. Students represented 61.1% of the patients. The hemoglobin profiles were homozygous SS (*n* = 14) and double heterozygous SC (*n* = 4). Most of the crisis (83.3%) occurred at night. Most of the patients (66.7%) came to the hospital before the sixth hour of crisis, one patient came by the 48th hour. Walking was the most self‐relief method tried by patients (67%). It was followed by a cold penile bath, attempted urination, body bath, and lastly lukewarm bath. Fourteen patients had a history of chronic intermittent priapism. The average pain intensity was 9.5 ± 0.9 with restlessness (33.3%) and crying (33.3%). Fifteen patients were treated upon admission with an intracavernosal injection of epinephrine, and three patients were first drained. Thirteen patients achieved remission immediately, while five patients required a second injection and only one had to be drained before remission. Tolerance was good. One patient had a borderline systolic blood pressure. One erectile weakness case was noticed and no cases of sexual impotence. Epinephrine by intracavernosal injection is an efficient treatment for acute priapism in sickle cell patients. Epinephrine, which has a good tolerance in pediatric and young adult patients, should be used in lieu of etilefrine due to its unavailability in areas where it is unavailable.

## 1 INTRODUCTION

Sickle cell anemia is the most common genetic disease in the World Health Organization (WHO) African region [1]. In Togo, the prevalence of the S gene is 16.1% with 1%–3% of major forms (SS, SC, or Sβthalassemia) [2]. Priapism (International Classification of Diseases N48.3) [3] is a well‐known urologic complication of sickle cell anemia. In fact, sickle cell disease is responsible for 65% of priapisms in children [4]. Priapism is defined as a prolonged painful and irreducible erection which occurs without any sexual stimulation. Its prevalence varies from 9.3% to 42% depending on the haplotypes [5, 6, 7, 8, 9]. The physiopathology is not clearly known. Sickle‐cell‐related priapism is a low flow priapism with two clinical forms: the acute priapism (AP), which is of a prolonged duration but usually isolated, and chronic intermittent priapism (CIP) which consists of repeated crisis of short duration priapism. The therapeutic strategy combines a pharmacological detumescence by intracavernosal injection (ICI) of alpha‐stimulants with the evacuating puncture of cavernous blood laterally at the penoscrotal or transbalanic junction [10, 11, 12, 13]. The use of etilefrine or undiluted phenylephrine has been preferred due to their purer alpha‐stimulating nature and for a better cardiovascular tolerance. Epinephrine (adrenaline) is a major alpha‐stimulant whose side effects are too severe, hence its low use in acute priapism (AP). In rare cases, it has been used in a strongly diluted solution. For the past 15 years, injectable etilefrine has not been available in Togo following the withdrawal of Effortil by Boehringer Ingelheim laboratories. The consequence is a difficulty in adequate traitement of the AP. Because of the availability of epinephrine in all hospital units in Togo, it has been used in the treatment of AP. This work reports the results we obtained after 1 year of use, the efficiency and tolerance of epinephrine in sickle cell AP.

## 2 PATIENTS AND METHOD

### 2.1 Study framework

This study was initiated by the sickle cell disease management section of the hemato‐oncology unit of the pediatric department of the Centre Hospitalier Universitaire (CHU) Sylvanus Olympio in Lomé.

The pediatric department of the CHU Sylvanus Olympio has a capacity of 170 beds and cribs spread over 10 hospitalization pavilions, including two continuing care pavilions. It is the national reference service for child healthcare in Togo. In the hierarchy of health structures in the country, the pediatric department is ranked at mid‐level. Each year, it receives around 9000 children from age 0 to 18 years, a third of whom are hospitalized. The hemato‐oncology unit has an oncology subunit and a hematology subunit that house the sickle cell management section.

#### 2.1.1 Description of the sickle cell disease management section

About 1500 sickle cell children have been followed there on an outpatient basis since 1996. The average number of admissions in each consultation (survey) is 40 sickle cell patients.

In general, children with sickle cell disease, whether monitored or not, admitted for AP are received in emergency in the general pediatric consultation room of the department.

#### 2.1.2 Overview of management for a child with acute priapism

Prior to being admitted, the patient tries to use self‐relief methods (these are called small measures to treat his priapism) and gives the specialist doctor a call when the priapism persists. With the doctor's agreement, the patient then goes to the hospital. Non‐monitored patients go directly to the hospital. Upon admission to the pediatrician office, the constants are taken in the waiting room. A short survey is carried out, followed by a quick clinical examination. A decision is made on the type of treatment. It is either an ICI of etilefrine or a puncture drainage followed by ICI of etilefrine. The material for the treatment gets prepared, the patient and the practitioner get ready also. Then the action follows. The patient is asked to walk for 15 min and then an assessment is made 15 and 30 min later. The effect of treatment is recorded and then the decision to end treatment or continue it is made.

### 2.2 Study methods

#### 2.2.1 Inclusion criteria

The patients included in the study were major sickle cell patients (SS, SC) admitted for a clinically estimated low flow AP.

#### 2.2.2 Non‐inclusion criteria

The following sickle cell patients are not included in our study: patients with CIP; or patients with an AP already treated by ICI before admission; or patients with an AP already operated in urology; or patients with an AP who has a history of cardiovascular disease; or patients with a high flow AP or a recent history of trauma.

#### 2.2.3 Study outline

We carried out a descriptive cross‐sectional study aimed at studying the epidemiological and clinical aspects of patients admitted for AP on the one hand and on the other hand the treatment by ICI of epinephrine for episodes of AP in the pediatric department of the CHU Sylvanus Olympio from January 1 to December 31, 2020. Our unit is a follow‐up unit for sickle cell patients located in a pediatric ward. Most of the patients are children, but some adults prefer to continue their treatment in this unit. The doctors working in the unit are qualified to treat sickle cell patients in general. This unit has expertise in the management of priapism. It was one of the first three centers in the world to experiment with ICIs. The children followed in the unit, who have become adults, know from the educational therapy that priapism is managed in the unit.

#### 2.2.4 Definitions adopted and parameters studied

AP is a prolonged painful, irreducible erection occurring without any sexual stimulation and developing for at least 4 h. CIP refers to repeated attacks of painful erection of short duration. The major sickle cell patient was a patient with an SS or SC or Sβthal hemoglobin profile. The (ICI) treatment is considered effective if there is relief of priapism or detumescence followed by a meaningful regression or relief of pain. Pain was rated on the visual analog scale (VAS).

#### 2.2.5 Description of the treatment

In all patients admitted before the 10th hour of AP crisis, the treatment is exclusive injection of epinephrine without prior drainage. A tight manual tourniquet is installed at the root of the penis. Then, using a 30 F needle mounted on a 1 ml syringe, the injection is done right under the balanopreputial sulcus in a cavernous body on the lateral aspect of the penis, with 0.25 mg (0.25 ml) of epinephrine for older children and adults and 0.12 mg for young children. A quarter of the vial of epinephrine (0.25 ml) is taken and then 0.75 ml of physiological saline is added.

In patients admitted after the 10th hour, drainage and epinephrine ICI is the treatment. A 19 G epi vein‐type needle (or 21 G for young children) is inserted into a cavernous body, on the lateral surface of the penis, right under the balanopreputial groove, and the blackish viscous blood is allowed to flow until red blood is obtained. A tight manual tourniquet is installed at the root of the penis. Then, using a 30 F needle mounted on a 1 ml syringe, injection is made at a different location from the drainage site but always immediately under the balanopreputial groove, 10 mg (1 ml) of etilefrine for the older children and adults and 5 mg (0.5 ml) for young children.

At the end of treatment, all patients were prescribed etilefrine tablets for 1 month, to be taken orally, once a day at bedtime.

#### 2.2.6 Data analysis

The data collected were entered into the Epi Data software. They were cleared and analyzed in the SPSS software (Statistic Package for Social Science).

## 3 RESULTS

### 3.1 Epidemiological aspects

The 20–24 years age group was the most represented in our series (*n* = 8). There were three patients in the 5–9 years old group, one patient in the 10–14 years old group, three patients in the 25–29 years old group, and three patients in the 30–34 years old group. The average age of the patients was 21.7 ± 7.7 years with ranges of 8 and 32 years. Two patients were married and had children, 12 patients were single without children, and four were children. The majority of patients (*n* = 11; 61.1%) were students. There were two students, two workers, two traders, and a tailor.

Half of the patients had reached secondary level 2 (high school). Four had a primary level of education, three a secondary level 1 (middle school), and two had a university level of education.

### 3.2 Clinical aspects

The majority of patients had known major sickle cell disease for more than 10 years (*n* = 13; 72.2%). Four patients (22.2%) were known to have sickle cell disease between 5 and 10 years and only one patient (5.6%) had been known to have sickle cell for less than 5 years. The patients' hemoglobin profile was SS hemoglobin in 14 cases (77.8%) and SC hemoglobin in four cases (22.2%).

Five patients suffered from AP for the first time. This was the second episode of AP in four patients, the third episode in two patients, and the fourth episode in two patients. Five patients were over their 100th episode. The first episode occurred at an average age of 20 ± 8 years. The age of onset of the first episode was recorded in 14 patients. The first episode occurred between 5 and 9 years in two patients, between 10 and 14 years in one patient, between 15 and 19 years in five patients, between 25 and 29 years in three patients, and between 30 and 34 years in three patients.

The majority of episodes (*n* = 15; 83.3%) occurred at night. Three patients had their priapism crisis during the day. The majority of patients (66.7%) had come to the hospital before the sixth hour after the onset of priapism and one patient had come by the 48th hour. Two patients were admitted within 3 h of onset. Ten patients between the fourth and sixth hour. One patient between the seventh and ninth hour and three patients between the 10th and 12th hour. A patient between the 16th and 18th hour and a patient between the 24th and 48th hour. In first intention, walking was the most small measure that most patients resorted to (67%). It was followed by cold penile bath (*n* = 2), then the use of etilefrine tablets (*n* = 1), then attempted urination (*n* = 1), followed by body bath (*n* = 1), and lastly lukewarm bath (*n* = 1).

In second intention, five patients walked, two patients ran, two patients walked, one patient had bathed, one patient had a lukewarm penile bath, and one patient consulted a nurse who put an ice bag on the penis.

AP had occurred in 14 patients against as a CIP and isolated AP in four patients. The CIP had been evolving for an average of 3.9 ± 2.9 years. Half of the patients had already had more than 10 episodes of CIP.

All patients had pain greater than 7/10 on the VAS upon admission. The average pain intensity was 9.5 ± 0.9. The majority of patients (72.2%) had pain rated at 10/10. Pain was 7/10 in 5.6%, 8/10 and 9/10 in 11.1% of patients.

### 3.3 Therapeutic aspects

Treatment was initiated between the fourth and sixth hour after the onset of AP in the majority of patients (66.7%). Nine patients were admitted and treated before the 10th hour. Of these nine patients, one was treated between the seventh and 10th hour, four patients between the 11th and 29th hour, and one patient after the 29th hour.

Of five patients admitted after the 10th hour, two received epinephrine ICI without prior drainage and three were first drained before epinephrine ICI. All patients admitted for the first 9 h were treated with epinephrine ICI from the start. The majority of patients (*n* = 13; 72.2%) had a detumescence 30 min after the first injection of epinephrine. Five patients required a second epinephrine ICI due to persistence or worsening of priapism (Table 1).

**TABLE 1:** Distribution of patients by the effect of first intracavernosal injection (ICI) of epinephrine

	Effect 15 min after ICI		Effect 30 min after ICI	
	Number	Percentage	Number	Percentage
Full detumescence	9	50	13	72.2
Partial detumescence	4	22.2	1	5.6
No change	4	22.2	3	16.6
Worsening	1	5.6	1	5.6
Total	18	100	18	100

Upon admission, on VAS 13 patients had pain intensity 10/10, two patients had pain intensity 9/10, two others 8/10, and one patient had pain intensity 7/10. Fifteen minutes after ICI, one patient had pain intensity at 10/10, one patient at 8/10, two patients at 7/10, one patient at 6/10, three patients at 5/10, one patient at 4/10, six patients at 3/10, one patient at 2/10, one patient at 1/10, and one patient at 0/10. Thirty minutes after ICI, one patient had pain at 10/10, two patients at 8/10, one patient at 6/10, one patient at 5/10, one patient at 2/10, five patients at 1/10, and seven patients at 0/10.

Fifteen patients (83.3%) were initially treated with epinephrine ICI. In the remaining three patients (16.7%), the epinephrine injection was preceded by a puncture drainage of the cavernous bodies. The tolerance of the first epinephrine ICI was good. Two patients had systolic blood pressure between the 90th and 95th percentiles and one patient had diastolic blood pressure in the same range. Restlessness (33.3%) and crying (33.3%) were the most observed general signs (*n* = 6, each 15 min later and *n* = 4, 30 min later). One patient had a rapid pulse at 15 min and then 30 min after the ICI. Burning‐like pain was noted at the injection site in three patients 15 minutes after ICI and in four patients 30 min after ICI.

Some patients (*n* = 5) required a second epinephrine ICI. Before this injection, one patient was at 10/10, two patients were at 8/10, one patient at 6/10, and one patient at 5/10. Fifteen minutes after ICI, the pain persisted in one patient (5/10), there was amendment in two patients (0/10 and 1/10) and regression in two others (3/10 and 2/10). Thirty minutes after the injection, there was an improvement in pain in four patients (three to 1/10 and one to 0/10) and persistence in the fifth (8/10). An amendment in the latter was noted after drainage and ICI. The second injection was given straight away without prior drainage in the five patients for whom the first ICI did not resolve the priapism. Fifteen minutes after the second injection the pulse was normal in all five patients. Four of them still had a normal pulse 30 min later and the fifth had a pulse at 100 per minute. One patient had a cut‐off systolic blood pressure between the 90th and 95th percentiles. No patient experienced dizziness.

The average duration of treatment was 95.5 ± 99.3 min with ranges of 1 h and 2 h 40 min.

### 3.4 Evolutionary aspects

Burning‐like pain at the injection site was recorded in two patients. All the patients were treated on an outpatient basis with an oral relay with etilefrine tablets for a period of 1 month. There was CIP managed at home in three patients. In 13 patients, there was neither AP nor CIP throughout the follow‐up treatment. Two patients had AP and were successfully treated with epinephrine. One patient presented with an erectile function disorder but no case of impotence was recorded.

## 4 DISCUSSION

### 4.1 Methodological bias

This study evaluated the treatment of AP with epinephrine ICI; it was a non‐randomized study. A randomized controlled study cannot be done in our center due to the availability of only one drug and due to the emergency nature of the event (priapism).

### 4.2 Epidemiological aspects

Our series was a small series which involved 18 patients, however the peak frequency reported between 20 and 25 years by Emond et al. [9] was confirmed. We had recorded patients aged 5–9 years as in the Lue series [14] but as many as in other age groups. PA seems rare between 10 and 20 years old, although from 5 to 45 years old, it can occur at any time in sickle cell patients.

### 4.3 Clinical aspects

All our patients are known sickle cell disease patients. It is important for them to make the decision of an early consultation for an urgent treatment. In fact, sickle cell patients were mostly informed of the possibility of this complication. It is usually brought to their knowledge during therapeutic education sessions and they know the risk of possible erectile dysfunction, so it stops them from wasting time and listening to the traditional explanations about priapism, which usually embarrass the patient and prevent him from consulting a doctor. In addition, the majority of patients have already had an AP so they knew the appropriate location and the treatment procedure.

The five patients with their first episode all consulted a doctor late between the ninth and 28th hour. Note that 66.7% of the other patients consulted early, especially before the sixth hour. This early consultation was the goal of therapeutic education in sub‐Saharan African countries, where consultation delays were frequent. Early consultation allows the healthcare team to have several treatment options; early management also allows rapid amendment and prevents long‐term complications. It is also known that when the AP episode is major with a persistence over several hours, permanent tissue damage occurs with deep ischemia, this defines a medical emergency related to partial or complete loss of erectile function [15].

In our unit, even if we go by the definition of AP with the bar lower than 4 h (definition which has become unambiguous [4, 16, 17]), our attitude toward patients corresponds to the description made by Broderick et al. [17] when they stipulated that for some sickle cell patients, an erection lasting more than an hour associated with pain corresponded priapism. This helps to avoid the consequences of penile ischemia and acidosis noted after the sixth hour.

The pathophysiology of AP is not yet fully understood, which explains the lack of a recommendation and a standardized protocol. The molecular mechanism which is being elucidated combines normal physiology and a dysregulation of erectile function, causing hemolysis: the pathogenesis of nitric oxide would indicate the use of phosphodiesterase type 5 inhibitors in the prevention of CIP. This remains subject to randomized phase III studies. More extensive studies must be continued to improve knowledge of this complication to establish formal recommendations.

### 4.4 Therapeutic aspects

Priapism is an urgent medical condition [17]. Achieving treatment objective is determined by the detumescence, the complications of which are conditioned by time. These complications are related to the duration of ischemia of the penis. Olujohungbe et al. reported that treatment around the fourth hour resulted in uncomplicated total remission [21]. Since the first work, the effectiveness of ICI has been constant. The first studies by Gbadoé et al. [10] had even reported detumescence in patients who had 5 days of AP, but classically after the 29th hour remission was no longer expected. In our study, the patient who arrived at the 48th hour had remission. Has there been a delay in tissue necrosis beyond the 24th hour in low flow priapism reported by Spycher and Hauri [18]? Would it have a different timeline depending on the individual? Since we only had one patient that arrived by the 24th hour, we cannot provide an answer to this question. Our study confirms, however, like in the other series [10] that treatment before the 29th hour with ICI was effective.

We believe, like Gbadoé et al. [10], that the management of an AP regardless of its duration must initially involve an ICI either of etilefrine or of epinephrine.

In most cases, the AP had occurred in the form of CIP. Identifying sickle cell patients at a risk for priapism would be important because sickle cell disease is a chronic disease that requires regular medical monitoring. The work of Kato et al. [19] identified patients with hemolysis biomarkers as those at risk. In other studies, patients with this complication had high hematocrit and hyperviscosity in the blood, however these were small series [17]. Low fetal hemoglobin level has also been implicated [20, 21] but conflicting results [22, 23] make it an unreliable predictor.

This study was initiated due to the unavailability of etilefrine, which was the main drug used in our department in the management of AP. The constant availability of epinephrine in the office and the continuous recording of cases of AP pushed us to write the protocol of epinephrine and conduct this study. In the literature, both molecules have been used. Thus, in the publications, French speakers preferred etilefrine while Anglo‐Saxons used epinephrine. According to the authors, these drugs were used in preparation or purely without preparation. Mantadakis et al. [24] and Virag et al. [25] recommended an injection without drainage before the sixth hour but in our series, three patients between the sixth and the ninth hour and two patients after the ninth hour were treated without drainage. Efficacy was obtained in the majority of cases without drainage, the latter occurring after failure of the injection. The tolerance of etilefrine has already been reported in several studies [24, 25] and in our department as well [10]. The non‐use of epinephrine despite its availability was probably due either to its safety or to concerns about its safety. In our study, despite the lack of comparison with etilefrine, the literature data allow us to state that epinephrine is as well tolerated as etilefrine. No serious side effects were recorded. The use of epinephrine is therefore now the reflex in the event of a priapism case in our department.

The use of etilefrine in preparation or diluted comes from the habit of using etilefrine which is not (usually used) diluted in our department, but the quantity used is between 0.5 and 1 ml. By trying to have the same volume, epinephrine is diluted with physiological saline for sufficient impregnation of the cavernous bodies.

A review of the literature on the management of priapism in Africa found that most publications came from Nigeria, but Togo was among the first teams to report ICIs. Sixty‐seven publications dealt with the subject, a dozen of which detailed the treatment [26, 27, 28, 29, 30, 31, 32, 33, 34]. Surgical treatment was used in most of the cases (Table 2).

**TABLE 2:** Distribution of African publications describing treatment by country, year, and type of treatment

	Title of the publication	Number, age of patients, and type of treatment
Nigeria [26] 2003	Priapism in southwestern Nigeria.	*n* = 16; mean age 20.4 years (range: 2.5–38 years). Needle aspiration + irrigation + injection of 2 ml of adrenaline solution (1 ml 1/1000 adrenaline in 200 ml saline) in both corpora cavernosa.
Nigeria [27] 2015	Management of priapism in adult men.	*n* = 18; median age 30 years (range: 17–60 years) . Glandulo‐cavernous shunt using the technique described by Ebbehoj.
Nigeria [28] 2016	Ischemic priapism in south‐east Nigeria: presentation, management challenges, and aftermath issues.	*n* = 15; mean age 30.5 years (range: 14–79 years). Intravenous hydration, aspiration, and irrigation. Glandulo‐cavernous shunt (Al‐Ghorab) in all.
Nigeria [29] 2000	Priapism in adult Nigerians.	*n* = 35; mean age 35 years (range 20–54 years). Immediate modified or conventional cavernospongiosus shunt.
Mali [30] 2014	Clinical and therapeutic aspects of priapism at CHU Gabriel Touré: study of 36 cases.	*n* = 36; mean age 17 years. Puncture of the corpora cavernosa.
Nigeria [31] 2009	Outcome of management of acute prolonged priapism in patients with homozygous sickle cell disease.	*n* = 54; mean age 20.56 ± 9.33 years (range 2.5–38 years). 70.37% surgically with Ebbehoj's cavernosa‐glandular shunt, 47.37% were treated conservatively.
Senegal [32] 2010	Acute priapism associated with sickle cell disease in Senegal: clinical, therapeutic features and risk factors for erectile dysfunction.	*n* = 22; mean age 19.5 ± 9.9 years (range 6 – 41 years). Corporeal aspiration with or without intracavernosal injection of sympathomimetics drugs and Al‐Ghorab shunt surgery.
Burkina Faso, Tchad, Niger, Gabon [33] 2000	Surgical treatment of priapism: experience of 56 cases in an African setting.	*n* = 56 (49 adults, 7 children). Surgical treatment: cavernous body shunt with unilateral transglandular cavernosalspongiosum shunt.
Nigeria [34] 1981	Priapism complicating sickle cell disease in Nigerian children.	Medical (*n* = 40). Analgesies, sedation. Pethidine, morphine, diazepam, dipyrone (novalgin), dihydrocedeine tartarate (DF 118) + heparinization i.v. NaHCO_3_ + blood transfusion i.v. NaHCO₃. Surgical (*n* = 5). Bilateral caverno‐spongiosum shunt or left cavernosaphenous shunt or bilateral cavernosa aspiration.

In summary, in practice, before the fourth hour, self‐relief methods (small means) are required. By the fourth hour, it becomes an AP situation, and the treatment becomes urgent. The goal of treatment is to correct compartment syndrome, establish normal blood flow in the penis, relieve pain, and prevent complications [14]. Before being admitted to the hospital, patients always started with small means. Those found in our study were identical to one in the other series, they included physical efforts, ways of cooling off the penis [6, 10]. The effectiveness of these methods has not been established [14]. The failure of these small measures led to consultation in the hospital. In this series, there is admission after administration of epinephrine. This treatment was effective in all patients. The effectiveness of epinephrine is well established. The method of administration and preparation were special features of our series. Remission in all of these patients without surgical treatment demonstrated the effectiveness of the treatment. This treatment was also well tolerated. The most troublesome sign was the burn at the injection site.

## 5 CONCLUSION

Priapism is a common urologic complication of sickle cell anemia. AP is a therapeutic emergency for which the first‐line treatment is medical. Pharmacological detumescence by ICI of alpha‐stimulants has been shown to be effective. The long used etilefrine in our service has never been continuously available in pharmacies in Togo. Ephedrine rarely used in this indication in our service has always been available. As the two molecules are from the same family and have similar pharmacological effects, this study was initiated in order to be more rapid in the treatment if the efficacy is obtained.

The efficacy of epinephrine has been confirmed in this work and offers an interesting alternative to the unavailability of etilefrine. Habit seems to be the factor that has delayed the use of this molecule in our department. However, the registered priapism cases should all be treated with ephedrine and patient registration should continue in order to communicate with a larger number of patients.

## CONFLICT OF INTEREST

There is no conflict of interest for the authors of this research.

## PATIENT CONSENT STATEMENT

All patients gave informed consent.

## ETHICS STATEMENT

The protocol was outlined and its application administered as described and in accordance with the advice of the consulted committee based on the revised Helsinki recommendations.

## AUTHOR CONTRIBUTIONS

*Study conception, data collection, analysis, exploration, and editing*: Koffi Mawuse Guedenon and Adama Dodji Gbadoe. *Study conception, data collection, analysis, and editing*: Mawouto Fiawoo. *Data collection and editing*: Djatougbe Ayaovi Elie Akolly and Fidèle Comlan Dossou. *Editing*: Etse Akpako and Balakibawi Esso.

## FUNDING INFORMATION

The authors received no specific funding for this work.

## Data Availability

The data that support the findings of this study are available from the corresponding author upon reasonable request.
